# Comparison of Neural Correlates of Reactive Inhibition in Cocaine, Heroin, and Polydrug Users through a Contextual Go/No-Go Task Using Event-Related Potentials

**DOI:** 10.3390/biology11071029

**Published:** 2022-07-08

**Authors:** Clémence Dousset, Christie Chenut, Hendrik Kajosch, Charles Kornreich, Salvatore Campanella

**Affiliations:** 1 Laboratoire de Psychologie Médicale et d’Addictologie, ULB Neuroscience Institute (UNI), CHU Brugmann-Université Libre de Bruxelles (ULB), 4 Place Vangehuchten, 1020 Brussels, Belgium; clemence.dousset@ulb.be (C.D.); hendrik.kajosch@chu-brugmann.be (H.K.); charles.kornreich@chu-brugmann.be (C.K.); 2 Substance Abuse Unit 73, CHU Brugmann, 4 Place Vangehuchten, 1020 Brussels, Belgium; christie.chenut@chu-brugmann.be

**Keywords:** event-related potentials, substance use disorder (SUD), cocaine, heroin, polydrug, inhibition, error-processing

## Abstract

**Simple Summary:**

Witnessing the current increase in the use of substances in society and considering the associated pervasive relapse rate, the management of addictions remains a significant challenge. The identification of biomarkers that are linked to specific profiles of consumption would allow a more targeted, and therefore, more effective care. In this view, the present study evaluates and compares the cognitive performance usually associated with substance use disorder—inhibitory control, attentional bias, and error detection—of heroin, cocaine, and polydrug users to matched healthy controls. Simultaneously, the addition of measurement of the modulation of brain activity during the task (event-related potentials technique) offers a reliable representation of the neuronal mechanisms underlying cognitive functioning. The results reveal substance-specific neural patterns of response, notably a more deleterious impact on polydrug use, and, despite nonsignificant results, suggest a more drastically affected cognitive functioning in cocaine users. Such evidence refines our knowledge of the specific mode of action of each substance. Ultimately, knowing their neural signature will lead to the implementation of more targeted interventions, thereby allowing specific needs to be addressed.

**Abstract:**

Recent global data indicates a worldwide increase in polydrug use associated with a shift from recreational to productive habits of consumption. Such non-responsible abuse of substances (alcohol, cocaine, heroin, etc.) is likely to lead to addictive disorders that are characterized by various neuropsychopharmacological effects. A main cognitive function involved in the onset and long-term maintenance of addiction is reactive inhibition, i.e., the ability to withhold a prepotent motor dominant response. In the present study, 63 (poly)drug user patients who were undergoing a detoxification program, in addition to 19 healthy controls matched for gender, age, and education, were subjected to a “contextual Go/No-Go task” with concomitant electroencephalography. Stimuli were superimposed on three contextual backgrounds: control (black screen), drug-unrelated (neutral pictures), or drug-related (pictures related to drug consumption). Of these patients, 23 were cocaine users (CU), 21 were heroin users (HU), and 19 were polydrug users (PDU). The main results showed that (1) at the behavioral level, more commission errors occurred with the PDU patients compared to the healthy controls; (2) at the neurophysiological level, specific alterations were found on classical event-related potentials that index reactive inhibition. Indeed, the higher rate of errors in the PDU group was subtended by both reduced amplitude and latency on the ∆N2 component and increased ∆P3 latency compared to controls. These data clearly suggest a more deleterious impact of polydrug use on inhibitory functions. In addition, our results provide evidence of reduced ERN amplitude in cocaine users, suggesting that impaired performance monitoring and error-processing may support impaired awareness, thereby preventing these patients from changing their behaviors.

## 1. Introduction

In order to cope with ubiquitous present-day social stressors (i.e., performance pressure), drug habits have expanded from recreational and occasional consumption to increasingly functional and productive drug use, including athletic performance enhancement, creativity enhancement in the arts, and cognitive enhancement in academic education [1,2,3]. According to the 2021 World Drug Report (a United Nations publication), between 2010 and 2019 (1) the number of opioid users nearly doubled in the global population, with a 65% rise in heroin use, which remains the main opioid in Europe; (2) among people entering treatment for cocaine use disorders in the European Union, more than two-thirds (79%) reported using cocaine in combination with heroin or other opioids [4]. These data are in keeping with a cross-sectional study of urine drug test results performed on more than a million patient samples in the United States: the use of opioids rose nearly 20-fold among cocaine users between 2013 and 2019 [5]. Thus, in a society of pervasive pharmacological reliance, this epidemiological evidence highlights an expansive and sharp increase in polydrug abuse. Hence, as the durability of non-responsible drug use raises the concern of subsequent dependence conditions, broader knowledge regarding the clinical impact of these polydrug habits becomes paramount.

Leveraging years of extensive research, the association between substance use disorders and cognitive impairments is now well recognized from both neuropsychological ([6], see [7] for a review) and neurophysiological perspectives (see [8] for a meta-analysis). Among all the observations made and the several theoretical models proposed to understand the underlying mechanisms of the development and maintenance of drug dependency (see [9] for a review), a specific imbalance in cognitive functioning stands out as a systematic pattern of a drug cycle: increased salience of substance-related cues paired with disabled inhibition of the dominant response [10,11,12,13]. Practically, sensory cues associated with patients’ chosen substance (i.e., visual, olfactory, or environmental cues) preferentially divert their attention and trigger an overtrained pattern of substance use—a consumption behavior that has become dominant over time—consequently undermining the patients’ ability to withhold their consumption in the presence of these cues. On a neurobiological level, this theory is linked with the impaired response inhibition and salience attribution (I-RISA) model conceptualized by Goldstein and Volkow, highlighting the implication of the prefrontal cortex as a core structure underlying this imbalance [14,15]. The I-RISA model has recently been greatly substantiated by both the meta-analyses of (1) Lueng et al. in 2017, revealing a small yet significant relationship between impulsivity and cognitive bias [16], and (2) Zilverstand et al. in 2018, who replicated the previous observations made regarding the alteration of both the salience and executive networks in addictive states [8,17,18,19]. Interestingly, the available evidence supports the central role of these particular functions throughout various stages of the addiction cycle: a transition from recreational drug use to dependency, craving, withdrawal, etc. [8,15], the impairment of which reduces the efficiency of clinical treatments, and accelerates the phenomenon of relapse [20]. It should be noted that several other cognitive dysfunctions, such as memory, metacognition, and decision-making, may play roles in treatment outcomes [7]. For instance, a lack of insight that reflects poor functioning of the interoceptive system is likely to lead to underestimation of the risks of abusive and hazardous consumption [21].

As stated by the consensus of the Neuroscience Interest Group within the International Society of Addiction Medicine (ISAM-NIG), fine-tuned knowledge of the multi-level determinants (neural, cognitive, and behavioral) of drug dependency needs to be addressed [20]. Interestingly, and in line with the previous statement, event-related potentials (ERPs) offer a reliable source of information regarding cognitive-related cerebral dynamics, as they have the special feature of allowing for the monitoring of cognitive-processing streams with high temporal resolution [22]. Technically, this approach allows the extraction of the truly event-related EEG signal from the superimposed background noise. In practice, the EEG activity is recorded concomitantly with the same experimental protocol that is repeated several times. Afterward, the recorded brain activity synchronized with the experimental protocol is averaged [22,23]. Based on the central limit theory, the signal that is stationary between trials gradually emerges from the noise as more trials are added to the average, while the randomly distributed noise decreases. The ERPs obtained are therefore a reliable reflection of the neural functioning of the cognitive abilities elicited by the chosen experimental protocol. In this view, the Nogo N2/Nogo P3 complex and the error-related negativity (ERN) component are classical ERP components that refer to inhibitory control and metacognition capacities, respectively [23].

Thus, in the context of substance use disorder, ERPs can reveal latent cognitive disturbances that are still inconspicuous at the behavioral level and, in this way, allow for the identification of various biomarkers [13,23]. Moreover, while the same behavioral pattern can be attributed to different cognitive impairments from one patient to another [24], ERPs have the potential to discern drug-specific modes of action and, in this way, promote more individualized and specific care by targeting specific processes that need to be rehabilitated [13,20,25]. Despite that, important ERP data are still missing, and a clear imbalance remains between the number of studies published on the various substances, notably with studies investigating the effects of heroin being clearly under-investigated in comparison with substances such as alcohol or cocaine [25,26]. This lack of empirical data prevents us from disposing of an exhaustive view of the neural signature of each specific substance, making the establishment of a differential diagnosis difficult.

In 2021, the Statistical Bulletin of the European Monitoring Center for Drugs and Drug Addiction reported that problematic use of cocaine and opioids accounts for most hard drug treatment requests [27]. In line with the previous statements, the main aim of the present study was to investigate the executive functioning, e.g., inhibitory control and selective attention, at the behavioral and the neurophysiological levels (using ERPs), of a population abusing the most recurrent substances (cocaine and heroin) and currently undergoing a detoxification program at the hospital. At the behavioral level, we expected that polydrug consumption would be associated with a higher number of inhibition errors due to the combined neurotoxic effects of cocaine and heroin [26]. At the electrophysiological level, decreased and/or delayed main ERP correlates of reactive inhibition; in other words, the Nogo N2, Nogo P3, and ERN, are expected to subtend this deficient behavioral performance [17,28].

## 2. Materials and Methods

### 2.1. Participants and Ethics Statement

Inpatients aged between 20 and 60 years who were diagnosed with heroin, cocaine, or polydrug dependence [29] and undergoing a two-week Drug Detoxification Program at Brugmann Hospital (Brussels, Belgium) were enrolled in the study. The exclusion criteria for participants included major medical conditions, neurological disorders, pregnancy, current consumption of drugs other than nicotine (assessed through urine screening), and a current DSM-V diagnosis of axis I disorders (other than drug dependence). The Brugmann Hospital ethics committee approved our study (Comité d’Ethique Hospitalier OM026 2015/121). All of the participants provided their informed written consent in accordance with the declaration of Helsinki.

The principal objective of the detoxification program was physical withdrawal. During their two-week stay, each patient received personalized medication and mainly benefited from living in a community aimed at finding a rhythm in their everyday behaviors (e.g., communicating, interacting with other patients, getting some rest, re-establishing a stable sleep cycle, partaking in sporting activities, etc.). In addition, support groups and an interview with a psychologist were offered, with the objective of creating opportunities for dialogue and eventually preparing them for discharge by informing them of the various post-treatment support options.

Among the N = 86 participants recruited and enrolled in this experiment, four patients exhibited an artefactual signal due to a poor signal-to-noise ratio, thus leaving 82 participants with analyzable EEG data who were included in the final sample used for the statistical analyses. The full characteristics of the group are reported in Table 1.

### 2.2. Procedure

The patients were free to leave the study at any time without having to justify their decision. The first several days were devoted to physical withdrawal. The patients were tested after showing withdrawal symptoms, approximately one week after admission.

They were asked to rate their urge to consume drugs according to a craving scale from 0 to 10, and to fill out the following questionnaires: the Liebowitz Social Anxiety Scale [30]; the Beck Depression Inventory (BDI-II) [31]; the State-Trait Anxiety Inventory (STAI-A and B) [32]; the Urgency Premeditation Perseverance and Sensation Seeking Impulsive Behavior Scale (UPPS) [33], the behavioral avoidance/inhibition (BIS/BAS) scales [34], and the Contemplation Ladder [35]. Additionally, an anamnesis and the Addiction Severity Index (A.S.I) [36] were performed to obtain general information (e.g., age and level of education) and specific information regarding their consumption history (e.g., the number of years that they had been consuming, their previous participation in treatment programs, and hallmarks of a family history of dependency), as well as the Mini-International Neuropsychiatric Interview (MINI) [37] short structured diagnostic interview, in order to control for any DSM-V axis-I psychiatric comorbidities.

### 2.3. The Go/No-Go Cognitive Task

**Figure 1:**
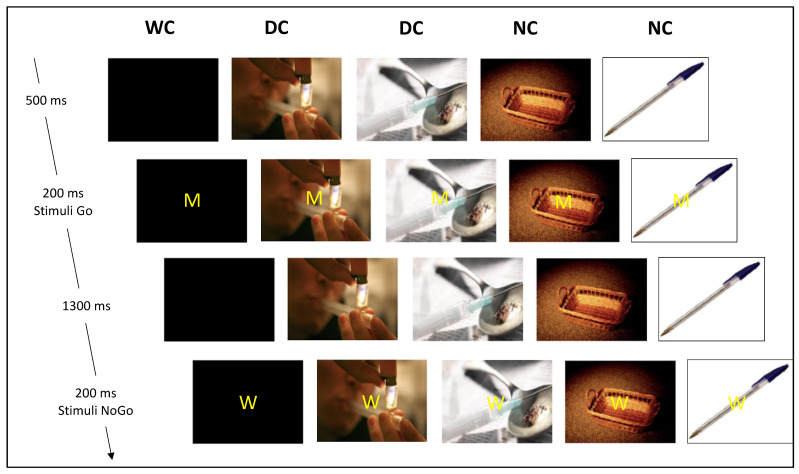
Illustration of the contextual Go/No-go task (without context (WC), neutral context (NC), and drug-related context (DC)).

All the patients were presented with a contextual Go/No-Go task that was developed in our laboratory for alcohol abusers [38]. For the present study, the cueing backgrounds were adapted to drug consumption. They were seated at a distance of one meter from a screen, which centrally displayed the letter M (“Go” trials; keypress with the right index finger, as quickly and accurately as possible) or the letter W (“No-go” trial; no button click required). The letters were superimposed on distinct pictorial backgrounds for each of the six blocks: two drug-related contexts (DC); two neutral contexts (NC); and two without context black screens (WC). Each block comprised 133 letters displayed in a semi-random order to avoid the consecutive presentation of two “No-go” letters, and divided into 93 “Go” (70%) and 40 “No-go” (30%) letters. Each block started with the presentation of a background screen (DC, NC, or WC; 500 ms) followed by the letter M or W appearing on this background screen for 200 ms, and then the initial background screen for 1300 ms. Thus, the patients had up to 1500 ms to press the button before the next letter appeared. Characteristics of the stimuli presentation are presented in Figure 1.

### 2.4. EEG Recording

Electroencephalograms (EEG) were recorded with a linked mastoid physical reference (M1, M2) in a 32-electrode Quick-Cap. The electrode positions included the standard 10–20 system locations and intermediate positions (Fpz, Fp1, Fp2, Fz, F3, F7, F4, F8, FC1, FC5, FC2, FC6, Cz, C3, C4, T7, CP5, CP1, CP2, CP6, T8, P7, P3, Pz, P4, P8, POz, O1, Oz, and O2). The EEG was amplified with battery-operated ANT^®^ amplifiers with a gain of 30,000 and a bandpass of 0.01–100 Hz. The ground electrode (AFz) was positioned between Fpz and Fz along the midline. The impedance of all the electrodes was maintained below 10 kΩ throughout the experiment. The EEG was recorded continuously at a sampling rate of 1024 Hz with ANT EEProbe™ software. Once acquired, for all conditions (DC, NC, and WC), a band-pass filter from 0.3 to 30 Hz was applied and response-locked epochs of 700 ms (200 ms before and 500 ms after the response onset) were created. A cutoff of 30 mV was used to define trials that were contaminated either by eye movements or muscular artifacts, which were detected offline and discarded from further analyses in order to analyze only the artifact-free trials.

**Figure 2:**
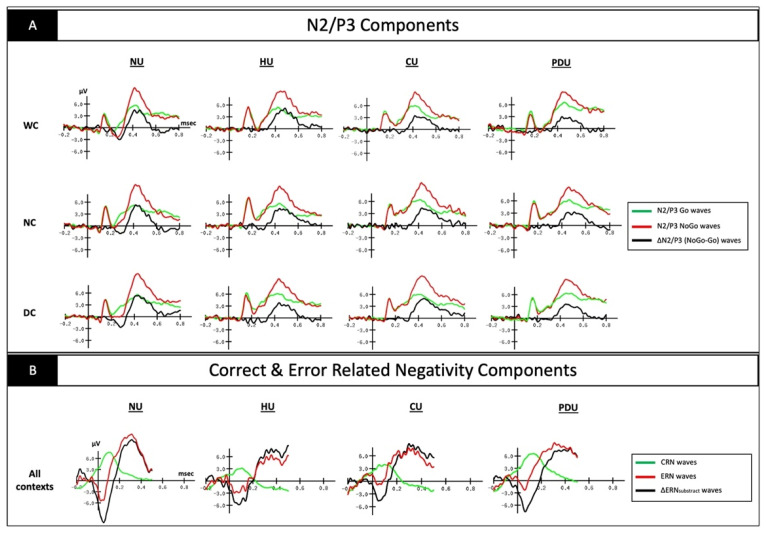
(**A**) Grand-averaged event-related potential waveforms obtained on Fz (as the best signal quality illustration) for hits on Go and non-hits on No-go trials during the Go/No-go task for all groups (non-users (NU), heroin-users (HU), cocaine-users (CU), polydrug-users (PDU)) and all conditions (without context (WC), neutral context (NC), and drug-related context (DC)). ∆N2 and ∆P3 were obtained by subtracting No-go waves from Go waves; (**B**) Grand-averaged event-related potential waveforms obtained on Cz (as the best signal quality illustration) for hits on Go and No-go trials during the Go/No-go task for each group, all contexts combined. ΔERN_subtract_ waves were obtained by subtracting ERN waves from CRN waves.

Only trials related to correct hits for targets, correct non-hits for non-targets, and hits for non-targets (commission errors) were included in the averages. Two parameters were coded for each stimulus: (i) the condition (DC, NC, or WC) and (ii) the type of response (keypress for targets, keypress for non-targets, and no keypress for non-targets). This coding allowed us to compute different averages of ERP: (1) the N2 component, identified as the largest negative value within the 200 to 400 ms interval after stimulus onset; (2) the P3 component, defined as the largest positive value within the 300 to 600 ms interval after stimulus onset; (3) the ERN produced subsequent to a keypress on non-targets (=errors); and (4) the correct-related negativity (CRN) produced subsequent to a correct keypress on targets. The averages were computed for each subject individually in the classical frontocentral cluster of electrodes (Fz, FC1, FC2, and Cz). All the analyses were carried out on the difference waveforms, which were calculated by subtracting the averaged Go-N2, Go-P3, and CRN waveforms from the averaged Nogo-N2, Nogo-P3, and ERN waveforms, respectively, thus giving rise to the ∆N2, ∆P3 [39], and ∆ERN_subtract_ [40] waves. Grand averages were then computed (see Figure 2A,B for illustrations).

### 2.5. Statistics

Statistical analyses were conducted using IBM SPSS Statistics^®^ 27.0 software (Armonk, NY, USA). Seven omnibus mixed analyses of variance (ANOVAs), using either Huynh–Feldt or Greenhouse–Geiser corrections when applicable, were computed with “Group” as a four-level between-subject factor as follows: heroin-users (HU; n = 21), cocaine-users (CU; n = 23), polydrug-users (PDU; n = 19), and non-users as healthy controls (NU; n = 19); and “Context” as a three-level within-subjects factor: drug-related context (DC), neutral context (NC), and without context (WC), for the behavioral (i.e., percentage of commission errors, percentage of correct hits, and reaction times) and electrophysiological (i.e., amplitudes and latencies of ∆N2 and ∆P3) data, respectively. Post hoc Bonferroni-corrected *t*-tests and supplementary analyses—multivariate and repeated measures ANOVAs—were used to accordingly disentangle significant main effects and interactions (significance level *p* < 0.05). Regarding the ∆ERN_subtract_ component, due to an insufficient number of averaged trials in each condition, all conditions were combined (DC, ND, and WC) and univariate omnibus ANOVAs—followed by post hoc analyses when applicable—were computed on N = 69 participants, with “Group” as the between-subject factor.

## 3. Results

### 3.1. Behavioral Data

The results of the 3 × 4 ANOVA conducted on the percentage of commission errors revealed a significant main effect of “Group” [F(3.78) = 4.390; *p* = 0.007; η^2^_p_ = 0.14]. Post hoc analyses revealed a significantly lower percentage of commission errors in the NU group (10.60 ± 6.78) compared to the HU group (22.92 ± 17.95; *p* = 0.023) and the PUD group (24.46 ± 11.01; *p* = 0.010), all conditions combined. No significant results emerged from the ANOVA conducted on the percentage of correct answers, nor on the reaction times (see Table 2).

### 3.2. Electrophysiological Data on the ∆N2 Component

Firstly, the results of the 3 × 4 ANOVA conducted on the ∆N2 component’s amplitude revealed a significant main effect of “Group” [F(3.78) = 4.341; *p* = 0.007; η^2^_p_ = 0.14]. Post hoc analyses revealed a significantly larger amplitude of the ∆N2 component in the NU group (−3.90 ± 2.35) compared to the CU group (−2.46 ± 1.56; *p* = 0.044), the PUD group (−2.09 ± 0.99; *p* = 0.008), and marginally to the HU group (−2.47 ± 1.56; *p* = 0.054), all conditions combined.

**Figure 3:**
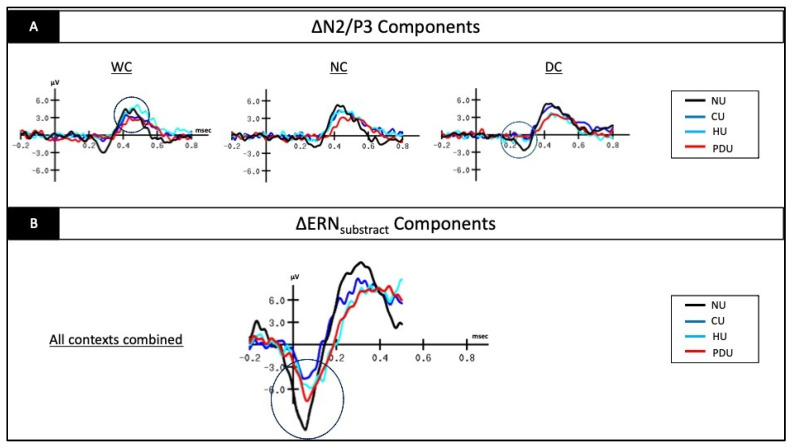
(**A**) Illustration on Fz electrode of the ∆N2 latency, ∆N2 amplitude and ∆P3 latency effects. (**B**) Illustration on Cz electrode of the ∆ERN_subtract_ amplitude effect.

Secondly, the results of the 3 × 4 ANOVA conducted on the ∆N2 component’s latency revealed a significant group*context interaction [F(6.156) = 3.308; *p* = 0.004; η^2^_p_ = 0.11]. On the one hand, the supplementary analysis computed using a repeated measures ANOVA revealed a significantly shorter latency in the DC condition (210.85 ± 28.97) compared to the WC condition (248.85 ± 57.22) in the PDU group (*p* = 0.041). On the other hand, the supplementary analysis computed using a multivariate ANOVA revealed significant main effects of group: (1) in the DC condition [F(3.78) = 15.973; *p* < 0.001; η^2^_p_ = 0.38]; (2) in the NC condition [F(3.78) = 10.101; *p* < 0.001; η^2^_p_ = 0.28]; and (3) in the WC condition [F(3.78) = 2.750; *p* = 0.048; η^2^_p_ = 0.96]. In the WC condition, post hoc analyses did not remain significant after application of the Bonferroni correction; meanwhile, they revealed the following: (1) in the DC condition, a significantly shorter ∆N2 latency in the PDU group (210.85 ± 28.97) compared to the HU group (285.29 ± 51.61), the CU group (305.11 ± 58.49), and the NU group (283.40 ± 36.67) (*p* < 0.001); (2) in the NC condition, a significantly shorter ∆N2 latency in the PDU group (212.71 ± 46.23) compared to the HU group (291.35 ± 49.09), the CU group (290.76 ± 53.96), and the NU group (281.39 ± 60.68) (*p* < 0.001) (see Figure 3A for an illustration).

### 3.3. Electrophysiological Data on the ∆P3 Component

While the 3 × 4 ANOVA conducted on the ∆P3 component’s amplitude did not reveal any significant results (*p* > 0.086), the 3 × 4 ANOVA conducted on the ∆P3 component’s latency revealed both a significant main effect of “Context” [F(2.156) = 3.829; *p* = 0.024; η^2^_p_ = 0.047] and a significant main effect of “Group” [F(3.78) = 3.477; *p* = 0.020; η^2^_p_ = 0.118]. Post hoc analyses indicated (1) a significantly shorter latency of the ∆P3 component in the WC condition (429.79 ± 50.45) compared to the DC condition (446.55 ± 48.72) (*p* = 0.042), all groups combined; (2) a significantly shorter latency of the ∆P3 component in the NU group (418.67 ± 42.33) compared to the PDU group (453.67 ± 37.30) (*p* = 0.019), all conditions combined (see Figure 3A for an illustration).

### 3.4. Electrophysiological Data on the ∆ERN_subtract_ Component

While the univariate ANOVA conducted on the ∆ERN_subtract_’s latency did not reveal a significant result (*p* > 0.179), the univariate ANOVA conducted on the ∆ERN_subtract_’s amplitude indicated a significant main effect of “Group” [F(3.65) = 3.822; *p* = 0.014; η^2^_p_ = 0.072]. Post hoc analysis indicated a significantly larger amplitude of the ∆ERN_subtract_ component in the NU control group (−11.34 ± 6.54) compared to the CU group (−5.35 ± 3.12) (*p* = 0.011), all conditions combined (see Figure 3B for an illustration).

## 4. Discussion

The aim of the present study was to highlight the cognitive deficits, i.e., inhibitory control and error detection, usually associated with substance use disorder, by comparing polydrug users, cocaine users, and heroin users undergoing detoxification to matched healthy controls at both the behavioral and the neurophysiological levels. In regard to the behavioral evidence, the results revealed impairment of the inhibitory skills in polydrug users, as well as in heroin users, through a significantly greater number of commission errors compared to controls. In concrete terms, the fact that polydrug and heroin users have more difficulties in restraining themselves from pressing No-go trials suggests substantial challenges in curbing automatic behavior, in holding back a motor action which is a self-regulatory capacity necessary to regain control over one’s consumption. These behavioral observations only partially reproduced the current evidence in the literature. On the one hand, contrary to what has been noticed in previous experimental studies, heroin users differed significantly from controls, while cocaine users did not. We should in fact have expected the opposite, as the meta-analysis of Smith et al. in 2014 indicates that the largest effect sizes for behavioral inhibitory deficits are found in cocaine users, whereas their results suggested that inhibition was probably not the core deficit in opioid users [26,41]. A potential explanation for this inconsistency could be the type of paradigm used [42]. Whereas an equiprobable Go/No-go task that requires little effort may not be challenging enough [43], the presently used Go/No-go task, in which the ratio for Go targets to No-go targets is 70% vs. 30%, should provide a sufficient degree of difficulty to observe significant differences between heroin users and controls. Furthermore, as response inhibition is not a stable trait and recovery of inhibitory control processes is a function of abstinence [42,44], another potential explanation could relate to the duration of abstinence. This may explain why recently detoxified heroin users currently undergoing a detoxification program differ significantly from healthy controls, while heroin users who have been abstinent for several months do not [45]. On the other hand, by examining the descriptive data of our sample, our observations replicate the existing evidence that polydrug use implies a larger deficit in inhibitory skills than use of a single drug [26]. Moreover, the following analysis of electrophysiological data (i.e., amplitudes and latencies of both N2 and P3 components) converge toward this same observation.

Firstly, compared to healthy controls, the results revealed a significant decrease in the ∆N2 amplitude in polydrug users, reflecting lower recruitment of neural resources in brain regions that underlie the detection and management of incongruence in a Go/No-go task context (i.e., occurrence of the non-frequent stimulus). As expected, this observation replicates those made in previous studies [28]. As anterior cingulate cortex (ACC) activity mostly reflects the amplitude of the N2 component [46], the present results reliably mirror the recurrently observed hypoactivation of the ACC in fMRI studies conducted on drug abusers [17,47]. More specifically, the evidence converges towards gray matter depletion in the ACC [48] linked to hypoactivation of both its rostroventral and caudodorsal subregions, with an added disruption of the interconnectivity between the two [49]. Moreover, the absence of significant differences between the different contexts (WC vs. NC vs. DC) coincides with the previously made fMRI observations that the ACC hypoactivity is not the result of attentional bias toward drug-related cues, but rather reflects a more general deviant cognitive process in drug users [50].

Nevertheless, while fMRI offers information regarding the activation level of brain regions, the ERP technique can add information regarding the timing of the onset of the cognitive process. In this case, our results indicate a significantly earlier latency of the ∆N2 component in polydrug users compared to all other groups, and even earlier in the drug-related context (vs. WC). Thus, despite attenuated subsequent management of incongruence due to hypoactivation of the regions in charge, the onset of the cognitive process of incongruence monitoring occurs earlier in a context, bringing into play visual substance-related cues. A possible interpretation is the presence of cue reactivity in polydrug users, indexed by the allocation of attentional resources toward stimuli related to the substance of interest. Interestingly, while this phenomenon is observable through an altered latency in the present Go/No-go task context, it has previously largely manifested itself by variation (increase) in the N2 component’s amplitude in oddball paradigms [28]. In a future perspective, it will be relevant to compare the electrophysiological observations made through different experimental paradigms. On a related note, it will be highly informative to conduct experimental studies by combining EEG and fMRI techniques, to make the most of their respective strengths—e.g., temporal and spatial resolution, respectively—in order to obtain the greatest amount of information about the underlying neural mechanisms [17].

Secondly, while the ∆N2 component can be seen as reflecting the early stages of the cognitive process setting up the inhibitory response [46], the subsequent P3 component is more a reflection of the actual motor aspect of this response, i.e., the restraint of the automatic motor action. In the present study, our results revealed significantly delayed latency of the ∆P3 component in polydrug users compared to control subjects. Even though both the ∆N2 and ∆P3 components reflect distinct aspects of the inhibitory process, they remain closely dependent on each other. In other words, an altered initial stage of the detection and monitoring of the conflict, and response selection reflected by the ∆N2 component characteristics (e.g., amplitude and latency) may have had an impact on the subsequent onset of the motor inhibition response reflected by the ∆P3 component [17].

Just as importantly, while the dysfunctionalities observed in the ACC and prefrontal regions underlie abnormal N2/P3 complex characteristics and explain inhibitory control deficits, it can also be linked to another ERP component that warrants attention: the error-related negativity (ERN). The ERN component is a reliable neurobiological marker that emerges after performance errors, thus reflecting the beginning of error processing as part of the action monitoring system [17,51,52]. Replicating findings in the literature and reflecting an impaired capacity of error processing, our results indicate a significantly reduced amplitude of the ERN component in cocaine abusers compared to controls [53]. Chronic use of stimulant drugs, such as cocaine, is associated with an increase in dopaminergic activity in the ACC and, through homeostasis, is likely to lead to down-regulation of dopaminergic receptors [54,55,56]. While several substances (i.e., alcohol, cocaine, opiates...) cause lower striatal dopamine D_2_ receptor binding [14,57], the study of Volkow et al. (1999) showed, in cocaine users specifically, a significant positive association between the amount of dopamine D_2_ receptors in the striatum and the amount of glucose in the frontal cortex: the fewer D_2_ receptors there are, the less glucose is available in the orbitofrontal cortex and anterior cingulate gyrus [14,58], leading to hypoactivity in the frontal cortical metabolism that underlies inhibitory control and error monitoring.

In conclusion, the evidence provided by the present study corroborates previous findings in the literature by revealing neural damage associated with cognitive disabilities found in substance abusers, i.e., disabled performance monitoring, poor inhibitory control, and deficient error processing. It is thought that defining features of addiction, such as the maintenance of drug-taking despite adverse consequences [47,56] and the relapse phenomenon, are associated with these specific cognitive deficits [20,28]. Indeed, error processing appears to be necessary to guide and adjust future behaviors, independently or not of conscious perception [54]. By being hyporesponsive to signals that their behavior is in error, patients may become overinfluenced by external stimuli associated with their substance of interest [56]. Such overreactivity to drug-related cues elicits a large amount of the available neural resources, thereby not leaving enough for the jeopardized inhibitory control to fight the habitual response behavior. In the long run, this entire compromised cognitive functioning leads to poor decision-making, stimulus-driven actions, and thus a more elevated risk of relapse [21,49,51]. Finally, corroborating the literature, our results suggest a more deleterious effect of poly consumption and cognitive functioning being more significantly impacted under the cumulative effect of several substances, in comparison to their individual incidence. Such a noxious synergistic impact stresses the importance of pursuing research on how to improve care in the field of addiction as poly-drug use increases significantly [4,5].

A main limitation of the present study is the absence of significant results between the PDU, CU, and HU groups in the ANOVA post hoc analysis (Bonferroni corrected), which may have been driven by a lack of power owing to a high degree of heterogeneity (SD) and, potentially, a too-small sample size. Nevertheless, the visually observed differences in ERP brainwaves remain highly informative as they reveal substance-specific patterns of response. For instance, despite the absence of significant results, visual analysis of the ERP waves clearly shows distinct profiles of response in the black screen context. Apart from any visual bias, a more pronounced attenuation of the ∆P3 amplitude in polydrug and cocaine users is observed, compared to heroin users and controls (see Figure 3A). As developed above, this difference is likely due to the known neuropsychopharmacological impact of chronic use of stimulants, such as cocaine, on the dopaminergic system [14,59]. Another explanation may lie in white matter integrity, as lower fractional anisotropy measures demonstrate axonal injury and demyelination in prefrontal regions [60,61]. In 2004, Lyoo et al. highlighted that the severity of deep white matter signal hyperintensities (WMH), regarded as localized demyelinated areas caused by vascular mechanisms and associated with cognitive performance [62], was greater in cocaine-dependent than in opiate-dependent subjects and likely due to cocaine’s vasoconstrictive effects [63]. Thus, compared to heroin users and controls, patients abusing cocaine (i.e., cocaine and polydrug users) exhibit an inhibitory process that is drastically affected by the neurobiological effect of this specific substance on the interacting mesocortical and mesolimbic networks [26,41,47]; the ∆P3 component is a neural marker of this deficit [17]. In this regard, the substance-specific patterns highlighted by ERP brainwaves support the view that each profile may require a specific rehabilitation method (i.e., compensatory or restorative learning approaches according to the neural damage distinctiveness), and in this sense is likely to lead to more individualized care [23]. Recently, phenotype-matched cognitive approaches using neuromodulation techniques and cognitive rehabilitation programs have been considered within the framework of stratified psychiatry [7,20].

## Figures and Tables

**Table 1:** Abbreviations: BDI, Beck Depression Inventory; STAI, State and Trait Anxiety Inventory; FNE, Fear of Negative Evaluation scale; BIS, Behavioral Inhibition System; BAS, Behavioral Approach System; NU = Non-users; HU = Heroin users; CU = Cocaine users; PDU = Polydrug users; NA = Not Applicable. Chi-square test, univariate analyses of variance with group (NU vs. HU vs. CU vs. PDU) as between-subject factor and the results of their respective post hoc analyses. The descriptive data are expressed as numbers, or mean ± SD. Significant results are highlighted in bold. * *p* < 0.05; ** *p* < 0.01; *** *p* < 0.001.

		Non-Users Healthy Controls n = 19	Heroin-Users n = 21	Cocaine-Users n = 23	Polydrug-Users n = 19	Statistical Data	Post-Hoc Analyses
Demographical and clinical data	Gender (male:female)	13:6	16:5	21:2	17:2	χ^2^(12) = 16.342; *p* = 0.176	NA
	Education (number of years)	11.32 ± 2.98	9.29 ± 2.83	10.91 ± 2.98	9.47 ± 3.20	F(3,78) = 2.325; *p* = 0.081	NA
	Age	37.89 ± 11.49	41.14 ± 9.01	36.83 ± 6.62	39.11 ± 8.85	F(3,78 ) = 0.903; *p* = 0.444	NA
	Drug consumption among family members (none:extended family:close family)	15:3:1	13:3:5	15:1:7	9:2:8	χ^2^(6) = 8.374; *p* = 0.212	NA
	Alcohol consumption among family members (none:extended family:close family)	16:3:0	6:7:8	14:2:7	9:2:8	χ²(6) = 18.136; *p* = 0.006 **	NU < HU = CU = PDU
	Number of past treatment programs	NA	2.00 ± 2.65	1.13 ± 2.36	1.37 ± 2.03	F(2,60) = 0.776; *p* = 0.465	NA
Psychological questionnaires	BDI-II	5.37 ± 8.31	16.29 ± 8.41	19.14 ± 12.35	20.63 ± 8.60	F(3,78) = 9.831; *p* < 0.001 ***	NU < HU = CU = PDU
	STAI-state A	44.21 ± 11.21	51.52 ± 10.97	58.00 ± 9.55	52.79 ± 9.05	F(3,78) = 6.242; *p* < 0.001***	NU < CU
	STAI-trait B	41.84 ± 9.45	53.24 ± 10.23	56.68 ± 9.55	54.89 ± 9.56	F(3,78) = 9.303; *p* < 0.001 ***	NU < HU = CU = PDU
	FNE	10.63 ± 6.08	14.90 ± 5.87	17.95 ± 6.13	14.68 ± 5.13	F(3,78) = 5.373; *p* = 0.002 **	NU < CU
	BIS	17.26 ± 5.22	19.57 ± 2.31	20.68 ± 3.40	20.11 ± 2.47	F(3,78) = 3.588; *p* = 0.017 *	NU < CU
	BAS drive	8.84 ± 2.50	9.00 ± 2.49	11.41 ± 2.84	10.32 ± 2.58	F(3,78) = 4.470; *p* = 0.006 **	NU < CU
	BAS fun seeking	11.79 ± 1.47	11.81 ± 1.86	12.64 ± 2.17	12.47 ± 2.06	F(3,78) = 1.084; *p* = 0.361	NA
	BAS reward responsiveness	15.95 ± 2.55	17.38 ± 2.27	17.18 ± 2.01	17.47 ± 1.71	F(3,78) = 2.085; *p* = 0.109	NA
	UPPS Total	103.32 ± 13.90	103.29 ± 8.38	107.82 ± 20.97	102.16 ± 13.81	F(3,78) = 0.584; *p* = 0.627	NA
	*Urgency*	30.89 ± 8.23	30.38 ± 4.21	30.55 ± 8.66	28.79 ± 6.10	F(3,78) = 0.338; *p* = 0.798	NA
	*Lack of premeditation*	22.41 ± 6.09	21.29 ± 4.55	25.50 ± 6.06	21.32 ± 4.77	F(3,78) = 2.861; *p* = 0.042 *	NU = HU = CU = PDU
	*Lack of perseverance*	21.74 ± 4.77	21.67 ± 3.89	21.86 ± 3.94	21.53 ± 3.47	F(3,78) = 0.025; *p* = 0.995	NA
	*Sensation seeking*	27.85 ± 9.02	30.19 ± 6.67	28.36 ± 10.57	30.53 ± 9.44	F(3,78) = 0.424; *p* = 0.736	NA
	Ladder	NA	8.10 ± 8.95	8.45 ± 1.22	7.95 ± 1.35	F(2,60) = 0.693; *p* = 0.504	NA
	∆Craving(pre-post)	NA	−0.45 ± 2.77	0.12 ± 2.04	0.00 ± 0.82	F(2,60) = 0.456; *p* = 0.636	NA
A.S.I	Medical status	0.74 ± 1.63	1.90 ± 1.95	1.96 ± 1.85	2.26 ± 2.18	F(3,78) = 2.376; *p* = 0.076	NA
	Employment and support	0.95 ± 2.07	4.10 ± 2.84	2.87 ± 2.24	4.47 ± 2.34	F(3,78) = 8.466; *p* < 0.001 ***	NU < HU = CU
	Drug use	0.00 ± 0.00	7.48 ± 1.25	7.61 ± 1.23	7.89 ± 1.05	F(3,78) = 263.45; *p* < 0.001 ***	NU < HU = CU = PDU
	Legal status	0.00 ± 0.00	2.67 ± 2.56	2.48 ± 2.69	4.00 ± 2.54	F(3,78) = 10.17; *p* < 0.001 ***	NU < HU = CU = PDU
	Family/social status	0.53 ± 0.84	3.62 ± 2.76	3.52 ± 2.21	4.53 ± 2.50	F(3,78) = 11.73; *p* < 0.001 ***	NU < HU = CU = PDU
	Psychiatric status	0.53 ± 1.61	3.81 ± 2.84	5.30 ± 2.36	4.53 ± 1.90	F(3,78) = 17.31; *p* < 0.001 ***	NU < HU = CU = PDU
M.I.N.I	Major depressive episode (MDE) (none:past:current)	16:3:0	9:9:3	7:10:6	5:11:3	χ²(6) = 18.297; *p* = 0.006 *	NU < HU = CU = PDU
	MDE with melancholic features (none:current)	19:0	19:2	20:3	16:3	χ^2^(3) = 3.123; *p* = 0.373	NA
	Dysthymia (none:current)	19:0	17:4	20:3	18:1	χ^2^(3) = 4.831; *p* = 0.185	NA
	Suicidality (none:medium risk:high risk)	18:1:0	14:5:2	16:4:2	6:8:5	χ^2^(6) = 18.093; *p* = 0.006 *	NU < HU = CU = PDU
	Manic episode (none:current)	19:0	19:2	19:4	16:3	χ^2^(3) = 6.974; *p* = 0.323	NA
	Panic disorder (none:current)	19:0	19:2	20:3	18:1	χ^2^(3) = 2.881; *p* = 0.410	NA
	Agoraphobia (none:current)	18:1	20:1	20:3	15:4	χ^2^(3) = 3.540; *p* = 0.316	NA
	Social phobia (none:current)	18:1	18:3	20:3	17:2	χ^2^(3) = 0.975; *p* = 0.807	NA
	Post Traumatic Stress Disorder (none: current)	19:0	20:1	19:4	16:3	χ^2^(3) = 4.957; *p* = 0.175	NA
	Alcohol abuse (none:current)	19:0	17:4	8:15	12:7	χ^2^(3) = 22.534; *p* < 0.001 ***	NU < CU
	Generalized anxiety disorder (none: current)	18:1	12:9	16:7	12:7	χ^2^(3) = 7.703; *p* = 0.053	NA

**Table 2:** Mixed analyses of variance (ANOVAs) with group (NU vs. HU vs. CU vs. PDU) as between-subject factor and context (WC vs. NC vs. DC) as within-subject factor, in addition to the results of their respective post hoc analyses. The descriptive data are expressed as mean ± SD. Significant results are highlighted in bold. Abbreviations: WC = without context; NC = neutral context; DC = drug-related context; NA = not applicable. * *p* < 0.05; ** *p* < 0.01.

		Non-Users Healthy Controls n = 19	Heroin-Users n = 21	Cocaine-Users n = 23	Polydrug-Users n = 19	Statistical Data Group * Context Interaction Group Effect Context Effect	Post-Hoc Analyses
Commission error rates (%)	WC	10.16 ± 7.52	24.94 ± 20.28	17.95 ± 12.10	24.14 ± 12.25	F(5.6,146.6) = 0.970; *p* = 0.445 F(3,78) = 4.390; *p* = 0.007 ** F(1.9,146.6) = 2.800; *p* = 0.067	NA **NU = CU < HU = PDU** NA
	NC	10.99 ± 7.28	22.86 ± 17.37	19.67 ± 15.52	25.80 ± 12.34		
	DC	10.66 ± 6.91	20.95 ± 17.90	16.58 ± 14.25	23.42 ± 11.81		
	Total	10.60 ± 6.78	22.92 ± 17.95	18.07 ± 13.24	24.46 ± 11.01		
Ommission error rates (%)	WC	98.44 ± 2.60	96.34 ± 4.69	95.27 ± 10.43	96.26 ± 5.37	F(4.1,108.0) = 0.843; *p* = 0.505 F(3,78) = 1.864; *p* = 0.143 F(1.4,108.0) = 2.463; *p* = 0.108	NA NA NA
	NC	99.01 ± 2.22	96.54 ± 4.12	98.20 ± 2.09	96.97 ± 4.21		
	DC	99.43 ± 0.73	96.03 ± 6.04	97.82 ± 2.80	97.68 ± 3.12		
	Total	98.96 ± 1.72	96.30 ± 4.25	97.10 ± 4.18	96.97 ± 3.83		
Reaction times (ms)	WC	370.11 ± 63.52	413.33 ± 68.55	381.50 ± 54.50	388.00 ± 70.18	F(4.6,120.2) = 0.845; *p* = 0.513 F(3,78) = 1.526; *p* = 0.214 F(1.5,120.2) = 2.607; *p* = 0.092	NA NA NA
	NC	376.51 ± 91.23	400.64 ± 70.38	373.15 ± 52.76	379.24 ± 55.16		
	DC	355.47 ± 53.12	400.00 ± 53.27	374.39 ± 56.67	382.10 ± 56.80		
	Total	367.37 ± 58.51	404.66 ± 61.82	376.35 ± 53.52	383.11 ± 59.28		

## Data Availability

The data that support the findings of this study are available upon request to the corresponding author.
